# Performance Characterization of GNSS/IMU/DVL Integration under Real Maritime Jamming Conditions

**DOI:** 10.3390/s18092954

**Published:** 2018-09-05

**Authors:** Ralf Ziebold, Daniel Medina, Michailas Romanovas, Christoph Lass, Stefan Gewies

**Affiliations:** 1 German Aerospace Center (DLR), Institute of Communications and Navigation, Neustrelitz 17235, Germany; daniel.ariasmedina@dlr.de (D.M.); christoph.lass@dlr.de (C.L.); stefan.gewies@dlr.de (S.G.); 2 BASELABS GmbH, Chemnitz 09126, Germany; michailas.romanovas@baselabs.de

**Keywords:** maritime navigation, GNSS, GNSS jamming, integrated navigation systems, Kalman filtering, Doppler velocity log, inertial sensors

## Abstract

Currently Global Navigation Satellite Systems (GNSSs) are the primary source for the determination of absolute position, navigation, and time (PNT) for merchant vessel navigation. Nevertheless, the performance of GNSSs can strongly degrade due to space weather events, jamming, and spoofing. Especially the increasing availability and adoption of low cost jammers lead to the question of how a continuous provision of PNT data can be realized in the vicinity of these devices. In general, three possible solutions for that challenge can be seen: (i) a jamming-resistant GNSS receiver; (ii) the usage of a terrestrial backup system; or (iii) the integration of GNSS with other onboard navigation sensors such as a speed log, a gyrocompass, and inertial sensors (inertial measurement unit—IMU). The present paper focuses on the third option by augmenting a classical IMU/GNSS sensor fusion scheme with a Doppler velocity log. Although the benefits of integrated IMU/GNSS navigation system have been already demonstrated for marine applications, a performance evaluation of such a multi-sensor system under real jamming conditions on a vessel seems to be still missing. The paper evaluates both loosely and tightly coupled fusion strategies implemented using an unscented Kalman filter (UKF). The performance of the proposed scheme is evaluated using the civilian maritime jamming testbed in the Baltic Sea.

## 1. Introduction

Maritime transport plays a key role in the global trade with nearly 80% of commodity volumes and 70% of commodity values being transported by sea [1]. The increasing transport volume leads not only to rapidly growing vessel dimensions but also to an increase of traffic densities especially in coastal areas and port entrances. Here reliable and accurate navigational information is required to avoid situations that could compromise the safety of the ship, crew, and the environment. Currently, Global Navigation Satellite Systems (GNSSs), and particularly GPS, are the main source for the provision of absolute position, navigation, and precise time (PNT) information for maritime navigation. A large number of systems and functionalities onboard a vessel, such as the Electronic Chart Display and Information System (ECDIS), the Automatic Identification System (AIS), and the Automatic Track Control just to name a few, are strongly dependent on the provision of accurate PNT information. Furthermore, as first conceptual studies and demonstration projects have been started with the aim to development fully autonomous vessels [2], the need for accurate and reliable PNT will increase even further in the future.

This dependency on GNSS has raised serious concerns on the vulnerability of the navigation process [3,4]. As the GNSS signals are very weak (≈–125 dBm) when arriving to the receivers on the Earth’s surface, they become relatively susceptible to possible radio interference. Currently, a noticeable increase in intentional and unintentional radio frequency interference (RFI) in GNSS bands is observed. The act of intentionally directing powerful electromagnetic waves toward a victim’s receiver aiming to deny its operations is called jamming [5]. Especially the availability of cheap jamming devices such as Personal Privacy Devices (PPDs) has been widely recognized as a real threat to GNSS applications. One of a typical jammer’s application scenario is to dodge the unwanted tracking, e.g., as used in illegal fishing or by the drivers working overlong hours. In Newark, NJ, USA, the operation of an aviation ground-based augmentation system was disturbed by a PPD jammer used by a truck driver, regularly passing a road close to the airport [6]. Just recently, an international measurement campaign studying radio frequency interference (RFI) in the L1/E1 and L5/E5a bands on a container vessel was performed [7]. This study reports how frequent RFI events occur, even making the GNSS service unavailable in some cases. Moreover, South Korea has also repeatedly reported jamming attacks from North Korea, heavily affecting the maritime transport [8].

The International Maritime Organization (IMO) recognized the importance of resilient onboard provision of PNT data, and the development of Guidelines for an onboard PNT (data processing) unit has been identified as supplementary and necessary. The German Aerospace Center (DLR) has developed a concept for such a PNT unit [9] and was actively involved in the development of a corresponding guideline at the IMO, which has been finally approved in June 2017 by the IMO [10]. The basic idea behind the concept of the PNT data processing unit is to develop a scalable approach for a combined and harmonized usage of all available onboard sensors using the methods of multi-sensor and information fusion.

In general, one can distinguish three main groups of solutions that can be used for mitigation of GNSS jamming. Within the methods of the first group, the impact of jamming is mitigated inside the GNSS receiver by techniques such as adaptive notch filtering, pulse blanking, or adaptive beamforming using a multi-antenna GNSS receiver [11,12,13]. Within the second group, alternative terrestrial radio navigation systems are employed to enable a position determination independently from GNSS. However, after the decommission of LORAN-C (eLoran) in the US as well as in Europe, no global operational backup exists anymore. For maritime application, the so-called R-Mode (R-Ranging) is currently being developed as a terrestrial backup system. Here existing signals of opportunity of globally available maritime infrastructure such as MF radio beacons and ashore AIS stations will be used as possible ranging sources [14]. A first experimental testbed for R-Mode will be established in the R-Mode Baltic project (2017–2020) in the western part of the Baltic Sea [15]. Finally, within the third group, the positioning information from the GNSS receiver is combined within a multi-sensor fusion scheme with independent onboard sensors such as inertial sensors, a speed log, or a gyrocompass. Such hybrid navigation systems enable PNT determination even in cases where GNSS information are either not available or not sufficient for a complete solution.

The present paper focuses on an example from the third group, where a classical inertial measurement unit (IMU)/GNSS sensor fusion scheme is augmented with a Doppler velocity log (DVL). Although the benefits of integrated IMU/GNSS navigation systems have already been demonstrated for marine applications [16], a performance test of such a multi-sensor system under real jamming conditions on a vessel is still missing. Typically, the GNSS outage is only emulated by switching the GNSS signals off instantaneously, whereas within a real jamming scenario a complete GNSS outage is only the final stage after a slow degradation of GNSS signal reception. The crucial point is the correct description of the GNSS measurement error statistics under jamming conditions. Whenever the GNSS noise model assumptions are violated, the Kalman filter (KF) could behave suboptimally and therefore could result in inferior performance compared to that which is often demonstrated using pure simulated data. Typically, it is expected that the jamming leads to an increased range noise and might even result in failures in signal tracking loops. Therefore, the characterization of GNSS pseudorange errors statistics under jamming conditions is a prerequisite of using an IMU/DVL/GNSS integration in a real jamming scenario.

The rest of the paper is organized as follows. In Section 2, a brief overview of existing work on jamming experiments and the application of hybrid navigation systems for maritime applications is given. The details of the applied GNSS/IMU/DVL unscented KF are given in Section 3 and are followed by the description of the experimental setup of the measurement campaign in Section 4. The results are presented and discussed in Section 5, and a summary and outlook for future work are provided in Section 6.

## 2. Previous Work

The emerging of safety-critical applications subject to accurate GNSS positioning and timing, such as autonomous cars or assisted landing, has urged the GNSS community into identifying jamming as one of the major menaces [17,18,19]. Thus, lately there have been numerous studies related to detecting and counteracting the impact of jamming attacks. In [20], the performance of a broad range of consumer grade GPS receivers under the interference of a low cost PPD is analyzed. The test was carried out for a static scenario within a confined space, where the performance of the receiver in terms of positioning accuracy and solution availability was analyzed for both the jamming-free and jamming scenarios of different intensities. Surprisingly, the tested receivers coped properly with the interference during the light jamming attack (with a jamming-to-signal ratio of 15 dB) with only a marginal influence on the quality of the position solution. However, during the severe jamming attack (jamming-to-noise-ratio of 25 dB), the availability of the position solution was reduced by more than 75%, while the positioning accuracy heavily degraded. It appears that jamming merely introduces additional noise in the measured pseudoranges, while the positioning degradation occurs under powerful interference due to the loss of satellite signal track and a consequent poor satellite geometry.

A series of works addressing the impact of jamming attacks on the maritime navigation has been recently presented. For example, a trial was conducted on the East coast of United Kingdom using a professional L1 band jammer [21]. This study evaluated the jamming impact on the safety of maritime navigation and the quality of on-shore services such as vessel traffic management. The lack of GNSSs triggers numerous alarms and failures of interfaces (like the ECDIS) on the bridge of the vessel, causing discomfort to a vessel crew that additionally needs to face the challenge of quickly reverting to traditional means of navigation. This study nicely underlines the necessity for a backup for GNSS-based positioning on board a vessel.

Subsequently, a jamming experiment was carried out in the north of Norway using a broadband (bandwidth ≈ 60 MHz) jammer, centered at GPS L1 frequency and affecting the GLONASS primary frequencies [22]. The main experimental finding was that GLONASS G1 tracking remained more resistant to jamming than GPS L1. This finding could, however, have been caused by the fact that GLONASS G1 frequencies lie at the edge of the reported jammer bandwidth, and the effective jammer-to-signal power in that band could already be significantly lower than for GPS L1. The primary output of the work was the suggestion for the maritime community to update their GNSS receivers to modern multi-constellation and multi-frequency ones. While this might be a solution for some type of jammers, currently, there are already PPD jammers on the market being able to jam all relevant GNSS frequencies.

One of the typical approaches to bridge short GNSS outages is to combine GNSS information with that from an inertial measurement unit (IMU). The inertial sensors measure the relative state of the object with respect to the inertial navigation frame and have distinct advantages, such as being self-contained, immune to interference, and highly dynamical. Unfortunately, these systems provide only incremental information and the integration outputs drift over time when no external reference is provided [23]. An early work [16] assessed the potential application of IMU/GNSS integration for maritime navigation using a loosely coupled KF. A collection of IMUs of different grade was employed to characterize the position drift over time of the hybrid navigation system when the GNSS service was artificially disabled. Although the performance of the inertial sensors was relatively good, the authors concluded that, despite the tangible advantages from the usage of IMU, inertial sensors still could not be considered as a primary backup to GNSS due to their fast position drift even when high-priced navigation sensors are employed. This statement is corroborated by our recent work [24], where the performance of a hybrid IMU/GNSS system in maritime applications was also evaluated, including modern affordable tactical grade MEMS inertial sensors.

Another sensor typically used in maritime applications is the Doppler velocity log (DVL). When mounted on a vessel, the sensor faces the sea floor and can provide very accurate velocity information of the vessel with respect to the sea floor [25] for scenarios up to a certain depth. The working principle of a DVL is rather simple. Firstly, an oscillating acoustic signal is sent out along each of the transducer axes. From the frequency shift between the emitted and the returned signal, the velocity along each of the transducer axis is determined. Despite being commonly mounted on marine vehicles ranging from large ships to small autonomous underwater vehicles (AUVs), there is a scarcity of academic resources concerning the specifics and functionality of DVLs [26]. The combined usage of DVL and IMU have been reported in a number of works [27,28,29] for the navigation AUVs, although only in few works has it been applied to merchant vessels [30], and none have reported the performance of a hybrid IMU/GNSS/DVL navigation system. In a previous work [31], the performance of the navigation solution fusing IMU, GNSS, and DVL was demonstrated on a maritime scenario. However, the performance characterization under actual GNSS jamming conditions has not yet been realized.

## 3. Methods

The proposed hybrid navigation system employs the recursive Bayesian estimation (RBE) framework to combine the outputs of several sensors in order to obtain a single navigation solution. The RBE methods deal with the problem of estimating the changing-in-time state of the system applying a priori information about the underlying system dynamics and the observations from the sensors. The probabilistic paradigm has been widely applied to navigation and tracking problems, given its ability to accommodate inaccurate models as well as imperfect sensors [32].

In general, any RBE cycle is performed in two steps:

**Prediction**: The a priori probability is calculated from the last a posteriori probability using the available process model.

**Correction**: The a posteriori probability is calculated from the a priori probability using the measurement model and the current measurements.

Among the multiple RBE methods, the Kalman filter (KF) is probably the most well-known solution for the navigation problem. The original KF provides an optimal solution when the models are linear and the probabilities are Gaussian. However, when dealing with nonlinearities in the models, as is often the case for positioning problems, nonlinear extensions of KF such as the extended KF (EKF) or the unscented KF (UKF) can be used. In the case of the EKF, the nonlinear models are linearized around the most recent state estimate, while in the UKF the probability distribution is approximated using a set of deterministically chosen (non-randomly sampled) points in the state space, where every sample is assigned a particular fixed weight. This set of points conserves the Gaussian properties of the distribution under nonlinear transformations [33].

Although the EKF has been for a long time considered as a *de facto* standard for tracking applications [34], the UKF has emerged as an alternative, claiming even higher accuracy and robustness for nonlinear models [35]. The UKF employs the statistical linearization techniques and implements the scaled unscented transform (UT), where carefully selected samples of the state are propagated through the actual nonlinear functions. Then, the mean and covariance are recalculated back from the propagated points, yielding more accurate results compared to a conventional linearization routine. Although in the presented work we have adopted UKF as a core estimation framework, similar results are expected if the estimation process would have been formulated with an EKF with sufficiently high update rate [36].

In general, the navigation filter is formulated as a nonlinear estimation problem for the system governed by the following stochastic models:

(1) $$\begin{array}{ccc} x_{k} & = & f\left(x_{k-1},u_{k},\nu_{k}\right) \end{array}$$

(2) $$\begin{array}{ccc} z_{k} & = & h\left(x_{k},\epsilon_{k}\right) \end{array}$$

where $x_{k}$ is the state vector of length *n*, $z_{k}$ is the vector of observations of length *m*, $u_{k}$ is the control input, $\nu_{k}∼N\left(0,Q_{k}\right)$ is a zero mean process noise vector, and $\epsilon_{k}∼N\left(0,R_{k}\right)$ is the observation noise vector. The $f\left(\;\cdot \;\right)$ and $h\left(\;\cdot \;\right)$ are the dynamic models for the state propagation and measurement model, respectively. Here the noises neither have to be additive nor have to be of the same dimensionality as $x_{k}$ or $z_{k}$.

In our work, the vector $x_{k}$ describing the state of the vessel in a 3D space can be expressed as

(3) $$x={\left[q^{T}\;v^{T}\;p^{T}\;b_{a}^{T}\;b_{g}^{T}\left(dt\;\dot{dt}\right)\right]}^{T}$$

where *q* represents the attitude quaternion from the vessel local to the the earth-centered, earth-fixed (ECEF) frames, *v* and *p* are the 3D velocity and position in the ECEF frame, and $b_{a}$ and $b_{g}$ are the accelerometer and gyroscope offsets. Finally, for tightly coupled IMU/GNSS integration schemes, the state has to be augmented with GPS receiver clock offset dt and clock offset rate $\dot{dt}$. Further, GNSS clock offsets can be added to the estimated state if a multi-constellation GNSS scenario is considered.

Effective and accurate attitude estimation can be considered as a key component of almost any higher performance navigation system. Within our implementation, we have explicitly addressed a challenge of attitude estimation by separating the attitude part (quaternion) and the vector part of the estimated kinematic state. In the present work, we chose a unit quaternion as the attitude parametrization due to its computational efficiency, low redundancy, and absence of singularity. The unit quaternion, although consisting of four numbers, is deprived of one degree of freedom due to its unit norm constraint and, therefore, requires a special treatment within the framework of the UKF [37]. Except for the interesting part of quaternion handling, the rest of the process model follows a classical strapdown inertial mechanization approach for higher performance systems [23] and is formulated in the ECEF frame.

There are several options to construct the measurement models depending on the configuration of the filter. For a *loosely coupled* approach, a snapshot least-square adjustment is applied to estimate the position and velocity of the vessel. In the case of a classical code-based GNSS positioning, a time-of-arrival concept is employed to determine the receiver position from the measured code pseudoranges of the satellites in view. The positioning principle is based on solving a geometric problem from the measured ranges to the set of visible satellites with known coordinates [38]. The ionosphere corrections are estimated using the Klobuchar empirical model, while the Saastamonien model is applied for estimating the tropospheric corrections. Similarly, the Doppler shift of the GNSS signals can be used to compute the velocity and the clock offset rate of the receiver.

Note that at least four satellites have to be visible in order to solve explicitly for both the position and the velocity in loosely coupled architecture. If there are fewer than four satellites visible at a given epoch, no solution can be found and the GNSS measurement step of the loosely coupled filter has to be skipped. An alternative approach, also known as *tightly coupled* integration, avoids the intermediate snapshot position and velocity solutions by applying the code or Doppler measurements directly in the measurement models of the filter. This strategy puts no inherent restrictions on the number of satellites available and even fewer than four available measurements could still be employed to constrain, at least partially, the possible drift of the estimated position. The same holds for the Doppler GNSS measurements, which can be combined with the GNSS code-ranges to enforce a radial velocity measurement over each available link. The mathematical model to relate the pseudorange ρ and the pseudorange rate $\dot{\rho }$ to the state estimate is as follows:

(4) $$\begin{array}{ccc} \rho^{j} & = & u^{j^{T}}\left(p+q⊗l_{GNSS}⊗q^{*}\right)+c\left(dt-dt^{j}\right)+Tr^{j}+I^{j}+\epsilon_{\rho^{j}} \end{array}$$

(5) $$\begin{array}{ccc} {\dot{\rho }}^{j} & = & u^{j^{T}}\left(v^{j}-v+q⊗\left(\omega \times l_{GNSS}\right)⊗q^{*}\right)+c\dot{dt}+\epsilon_{{\dot{\rho }}^{j}} \end{array}$$

where the superscript *j* refers to the jth GNSS tracked satellite, $u^{j}$ is the unit line-of-sight vector between the satellite and the receiver, $l_{GNSS}$ represents the baseline between the IMU and the GNSS antenna expressed in the vessel body frame, *c* stands for the speed of light, Tr and *I* gather the tropospheric and ionospheric effects, respectively, and ω is the angular rate of the vehicle expressed in the vessel body frame. The operation ⊗ refers to quaternion multiplication. For more details on quaternion conventions and a comprehensive explanation on quaternion algebra, the authors recommend consulting [39].

In the case of a loosely coupled integration, an equivalent position solution error covariance can be calculated by using the geometry matrix from the iterated LS solution and a corresponding weight matrix, where the weights are assigned following the assumptions regarding the quality of each link. A simplification, commonly used in practice (and which we actually assume within the equivalent design of the tightly coupled architecture) is that the measurements from the different satellites are uncorrelated. The measurement weighting matrix can then be constructed as an inverse of the diagonal measurement noise covariance matrix. As the GNSS Doppler measurements are usually of a relatively high quality, a constant noise assumption can be easily adopted with an equivalent circular covariance approximation for Doppler LS solution in a loosely coupled configuration.

Finally, the DVL measurement model can be considered as the X–Y velocity measurement in the coordinate frame of the sensor including the lever arm compensation with respect to the IMU. In addition to the actual 2D velocity measurement, a constraint along the body vertical axis of the vessel can be employed (velocity projection in the body frame) as one can assume the vertical velocity to be zero on average. The constraint has been implemented within the UKF framework as so-called “pseudo-measurement” by extending the true sensor measurement with a third component, setting the measurement to zero with some associated measurement noise. This vertical velocity measurement significantly decreases the vertical position drift by reducing it from being cubic in time to becoming quasi-linear in time. The measurement model for the DVL speed observation $v_{DVL}$ is expressed as

(6) $$v_{DVL}=q^{*}⊗v⊗q+\omega \times l_{DVL}+\epsilon_{DVL}$$

where $l_{DVL}$ refers to the baseline between the IMU and the DVL positions within the local ship frame.

Naturally, for lower-cost IMUs the navigation performance is strongly degraded due to a fast accumulation of the errors caused by sensor noises, biases, scale factor errors, etc. Moreover, for non-augmented IMU/GNSS system (e.g., a system without the magnetometer, a gyrocompass, or multiple GNSS antennas), the attitude and some of the inertial sensor errors become weakly observable, and the ability of the system to effectively estimate them is strongly conditioned on the dynamics of the vessel. For these reasons, the baseline observations (non-collinear vector measurements) were incorporated from three spatially distributed GNSS antennas to ensure that the attitude drift is constrained when baseline measurements are available. Recall that the attitude integration is the first integral of three subsequent integrals, which constitute the strapdown inertial mechanization and the attitude errors start to dominate the other error sources relatively fast. A baseline observation is considered to be valid if the RTK-based baseline determination between two antennas resulted in a fixed integer ambiguity solution; therefore, up to three baseline observations can be incorporated into the measurement model at each epoch depending on the quality of the RTK solutions. Here, baseline estimation is realized in a loosely coupled manner, following the methodology presented in [40]. Nonetheless, heading determination can be also tightly coupled to the positioning problem for a higher exploitation of redundant information [41]. The advantage of a direct baseline vector observation model is that the heading becomes observable even with a single observation of non-vertical baseline (i.e., the baseline non-collinear with the local Earth gravity vector). Note that both pitch and roll angles are effectively observable via the coupling of the position measurements with the Earth gravity and only the heading information cannot be directly determined with a single GNSS antenna for typically limited vessel dynamics.

## 4. Setup and Measurement Campaign

**Figure 1:**
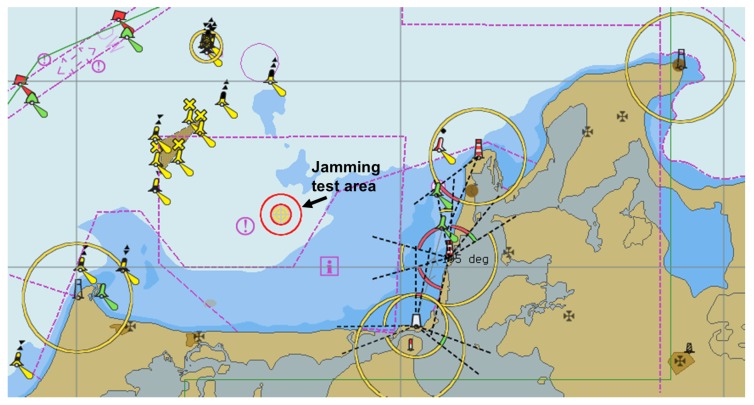
An overview of the civilian maritime jamming test area 10 km north of Peninsula Darß (54,5474 N, 12,8154 E).

**Figure 2:**
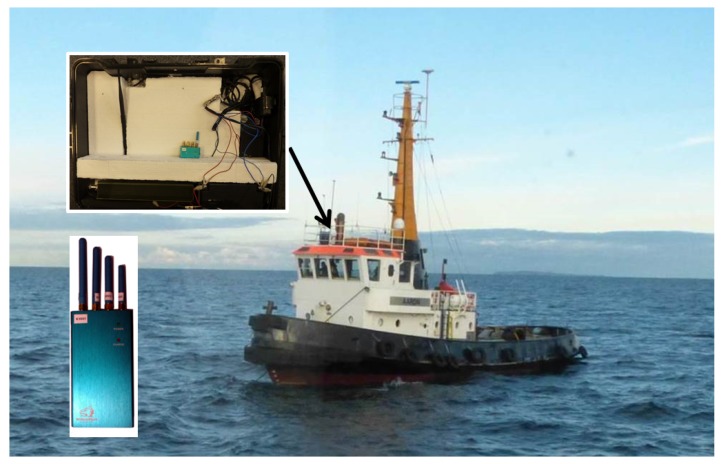
AARON with the jammer mounted on the monkey deck.

In order to enable experimental jamming tests under real life maritime conditions, the DLR in cooperation with the German Federal Network Agency has allocated a civilian maritime GNSS jamming testbed in the Baltic Sea. The test area is located approximately 10 km North of the Darß Peninsula (see Figure 1). The measurement campaign took place in November 2015. A PPD jammer (WolvesFleet 212G, output power: 2 W) was mounted on the monkey deck of the tugboat AARON (length 26 m, beam 8 m) (see Figure 2). During the campaign, the AARON was anchored in the center of the jamming test area, keeping a fixed position and heading. Due to waves (wave height: 1–2 m), the roll and pitch angle of the vessel varied significantly.

**Figure 3:**
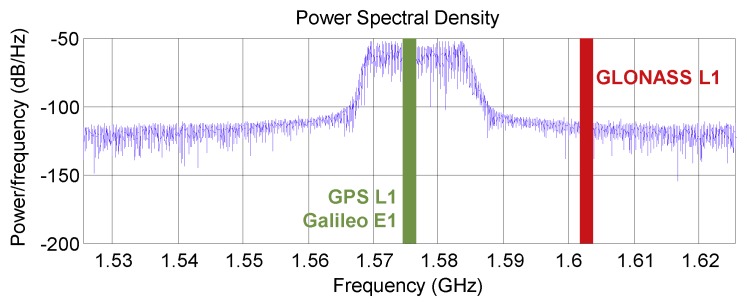
Power spectral density of the used PPD jammer.

The applied PPD jammer was sweeping a continuous wave signal with an update rate of ∼ 10 μs around the GPS L1 frequency covering a bandwidth of 17 MHz (see Figure 3) affecting both GPS L1 and Galileo E1 signal tracking, while GLONASS L1 mainly remained unaffected. This allowed us to use GLONASS for the calculation of a Precise Point Positioning (PPP) reference trajectory using RTKLib software [42] even in the direct vicinity of the jammer.

**Figure 4:**
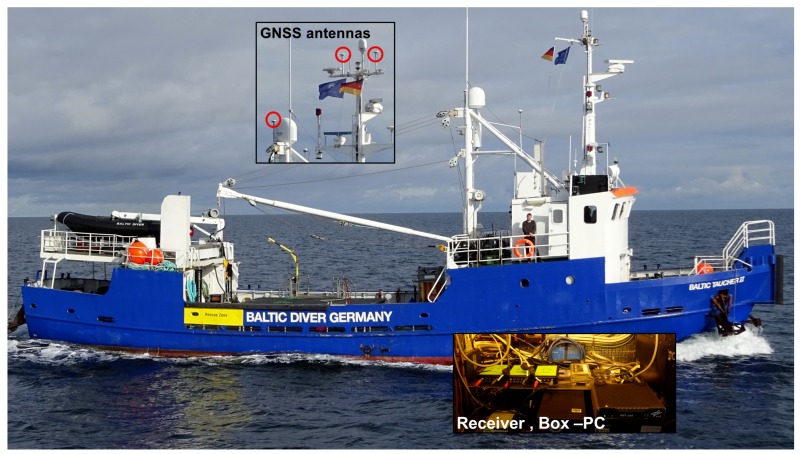
BALTIC TAUCHER II with the mounting of the antennas.

The measurement equipment was installed on BALTIC TAUCHER II, the multipurpose research and diving vessel (length 29 m, beam 7 m, see Figure 4), which was navigated around the tugboat AARON with a maximum speed of 8 knots and a distance to the jammer varying from ∼50 m to 4000 m. The vessel was equipped with three separate dual frequency GNSS receivers (type: Javad Delta, antenna type: navXperience 3G+C maritime), a FOG IMU (type Imar IMU FCAI, list of specifications at [43]), a gyrocompass, and a DVL (type: Furuno DS 60, list of specifications at [44]). All relevant sensor measurements were provided either directly via Ethernet or via serial to Ethernet adapter to a Box PC where the measurements were processed in real-time and in parallel stored in a SQlite3 database along with the corresponding time stamps. The described setup enables record and replay functionality for further processing of the original sensor data. The system consists of a highly modular hardware platform and a Real-Time software Framework implemented in ANSI - C++ as described in [45].

## 5. Results

Within the first part of this section, the results of the lab jamming experiment are shown. The experiment was used to investigate the validity of the adaptive range noise models in jamming scenarios. In the second part of the section, we demonstrate the performance of the hybrid IMU/DVL/GNSS navigation system under real jamming conditions using both the classical GNSS measurement noise model as well as the adaptive noise model, as described in Section 5.1.

### 5.1. Lab Experiment

**Figure 5:**
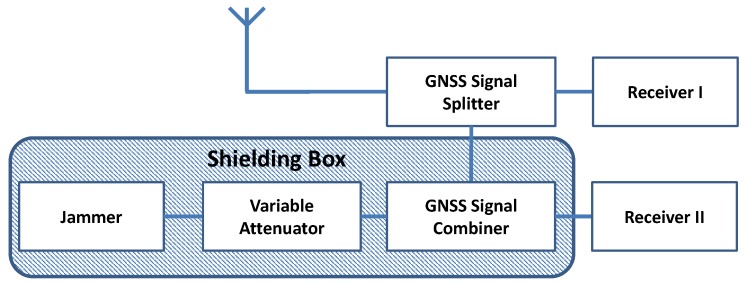
A schematic overview of the setup used in laboratory conditions.

For the lab experiment, an antenna receiver setup identical to the one of the maritime jamming measurement campaign described in Section 4 was used. The antenna has been mounted on the roof of the DLR office building in Neustrelitz (Germany). In Figure 5, the schematic overview of the experimental setup is given. One receiver (type Javad Delta) was acting as a reference using only the original GNSS signals from the antenna. As an input for the other receiver, a combined signal from the same GNSS antenna and the jammer was used. The antenna output of the jammer was connected using a HF cable, and an attenuated jamming signal was merged with the original GNSS signal. The jammer together with the attenuator and the GNSS signal combiner were placed in a shielding box ensuring that no measurable jamming radiation could be detected outside the laboratory. For each experiment, a 48 h (February 2017) measurement data with a 2 Hz update rate have been recorded. Two jamming scenarios were investigated with a total attenuation of 53 dB (Scenario A) and 42 dB (Scenario B), respectively.

**Figure 6:**
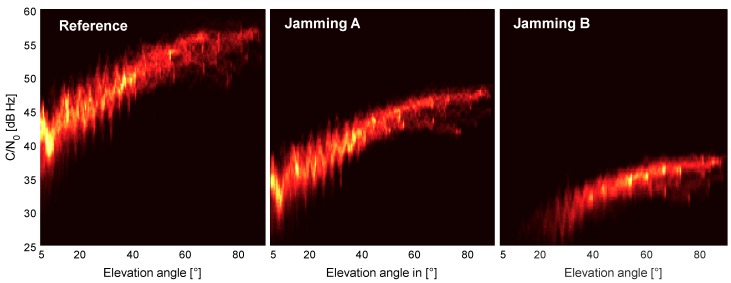
2D histogram plot of carrier-to-noise density ratio (CN0) versus elevation angle without jamming (**left**); for Scenario A (**middle**) and Scenario B (**right**).

In Figure 6, the impact of the jamming signal on the correlation between the carrier-to-noise density ratio (CN0) and the elevation angle is shown. For the unjammed reference, the mean of CN0 values varies approximately between 40 dBHz @ 5${}^{\circ }$ elevation angle and 57 dBHz @ 90${}^{\circ }$ elevation angle. For Scenario A, the 2D density distribution is shifted by approximately –10 dBHz, and fewer low elevation satellites are tracked. For Scenario B, the 2D density distribution is shifted again by approximately –10 dBHz (compared to Scenario A) and mainly higher elevation satellites remain visible. This corresponds fairly well with the difference of 11 dB of the attenuation of the jamming signal between Scenarios A and B. In Scenario B, only satellites above ∼30${}^{\circ }$ elevation are tracked by the receiver, resulting in only 4–5 satellites at each point in time. From the comparison of the three graphs in Figure 6, one can deduce that, for that receiver, a rather soft CN0 threshold for the tracking of the satellites exists, which lies between 25 and 30 dBHz. In the absence of jamming, low-elevation satellites show a signal strength gain of approximately 10 dBHz, compared to the signal strength under the effects of a jamming attack.

In order to determine the pseudorange statistics, the antenna position as well as the receiver clock offset of the reference was determined by a static precise point positioning (PPP) calculation with the RTKLib software [42] in post-processing using precise satellite orbit and clock information from the IGS service. The pseudorange errors were calculated as the difference between the expected and the observed ranges using broadcast ionospheric and tropospheric corrections (the same corrections which have been used within the filter design in Part 2 of this section) while taking into account the receiver clock offset from the PPP solution. The obtained data were binned according to the associated CN0 as measured by the receiver or to the elevation values, and for each bin a standard deviation was determined. Note that in this simplified approach we focus only on the standard deviation of the distribution and ignore the non-zero mean offset. However, pseudorange biases, which vary within the evaluated time span of 48 h, ultimately will also contribute to the calculated standard deviation.

**Figure 7:**
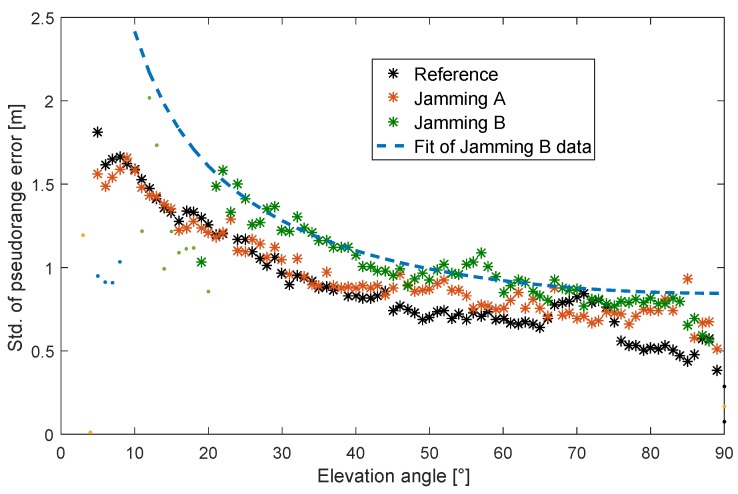
Measured dependency of the pseudorange error statistics (standard deviation) on the elevation angle in the lab experiment.

**Figure 8:**
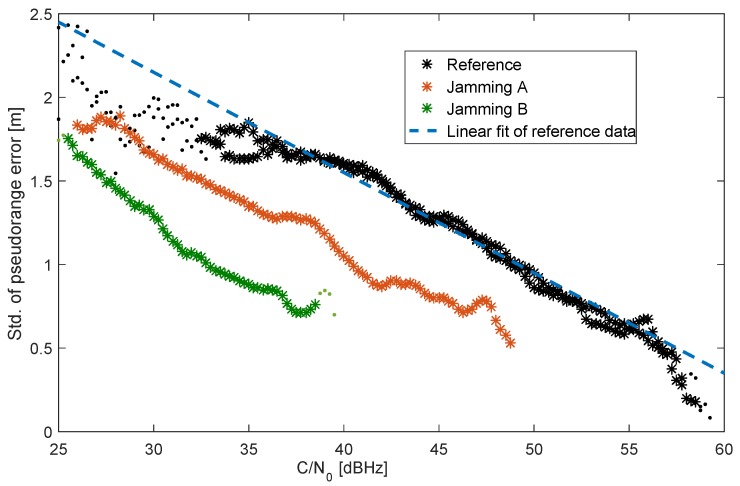
Recorded dependency of the pseudorange error statistics on the receiver reported CN0 value for lab experiment.

In Figure 7, the standard deviation of the errors is plotted against the elevation angle for both the original (no jamming) and for the two jamming scenarios. The crosses mark data points with an underlying test statistic including more than 1000 pseudorange errors, and the small dots mark data points with a sample population fewer than 1000 data points for that bin. The unjammed reference data show expected behavior, with increasing standard deviation for decreasing elevation angles. The measurements affected by the jamming signal show comparable behavior with fewer measurements for low elevation angles but only slightly overall increased standard deviations. Interestingly, the effect of the jamming signal on the standard deviation of the pseudorange measurements is rather minor, and this seems to be counterintuitive compared to the expected behavior of a typical receiver in the presence of a jammer. For the highest jamming signal power, only an increase by a factor of about 1.3 for the standard deviation can be observed. As an overbound for all data, including both jamming scenarios, the following functional dependence of the standard deviation σ and the elevation angle α was determined (see Figure 7):

(7) $$\sigma =A\times {\left(\sin\left(\alpha \right)\right)}^{-B}$$

with A=0.85 m and B=0.6. Elevation-dependent functions have often been applied in geodetic applications for expressing the expected noise of GNSS observations [46,47]. Finding the unknown parameters *A* and *B* constitutes a nonlinear regression problem, solved here by applying a least-squares adjustment. In Figure 8, for the same measurements as in Figure 7, the standard deviation as a function of the CN0 is shown. Interestingly, in the absence of the jammer, the reference data show an almost linear dependency on CN0. A linear fit leads to a slope of a=-0.06 m/dBHz and an offset of b=3.95 m. This linear dependency is different from a suggested exponential dependency as reported in literature [48,49]. To some extent, this could be explained by a new firmware of the receiver and a different antenna used compared to our previous work [49]. Comparing the curve for Scenario A with the scenario without jamming, mainly a parallel shift towards smaller CN0 values without a significant increased pseudorange error can be found. This finding is consistent with the analysis of Figure 6 and Figure 7. Furthermore, the curve for Scenario B is mainly shifted towards smaller CN0 values; it also starts at slightly higher standard deviations for the pseudorange error (the right-most end of the line starts at a higher error value with increasing jammer power), and error deviation increases faster with decreasing CN0 values.

Summarizing the results of Figure 8, one can conclude that, for the used receiver, the functional dependency of the standard deviation of the pseudorange error on the carrier-to-noise density, as is observed for the unjammed scenario, is rather an indirect correlation. The receiver tracking noise error, which should strongly depend on CN0, is small compared to other dominating errors, such as iononspheric, tropospheric, and multipath errors, at least for CN0 > 40 dBHz. For the usage of pseudorange measurements within a tightly coupled KF, this leads to an unexpected conclusion that a CN0-based weighting scheme, calibrated for the unjammed scenario, could result in overestimation of the pseudorange errors in the vicinity of a jammer.

Interestingly, the main impact of the applied sweeping PPD jammer on the used geodetic receiver is the reduction of the number of tracked satellites, while the pseudorange error statistics is only slightly effected. As expected, the receiver’s reported CN0 directly correlates with the applied jamming signal strength. Therefore, the dependency of the CN0 on the elevation angle is a good candidate for an indicator of the presence of a jamming signal in the environment. Of course, as the CN0 dependency is setup-specific, the relationship between the reported CN0 and the elevation angle has to be determined for each sensor configuration separately. The main motivation for the lab experiment was the determination of an appropriate pseudorange variance model in the presence of a jammer. One can conclude that, first of all, the constant noise model as the simplest possible model with a standard deviation of 2 m and uncorrelated observations is a good candidate for application in the hybrid navigation filter. Furthermore, the CN0-based variance model would not be significantly superior to a constant noise model as it overestimates the pseudorange errors in the presence of a jammer. Finally, the elevation-based GNSS measurement model seems to be a better candidate than the CN0 model in the case of the jamming as the reported noise statistics depends only slightly on the jamming power.

Still, an optimal pseudorange variance model could include both the dependence on elevation angle and the CN0. Such a model could in general be determined with an experimental setup used in this lab experiment but would require a far more detailed analysis and will be a subject of future work.

### 5.2. Results of the Maritime Jamming Campaign

#### 5.2.1. GPS Single Point Positioning Results

**Figure 9:**
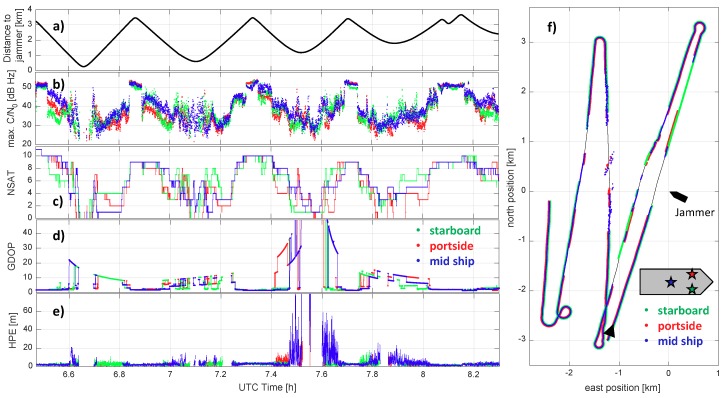
An overview of GPS positioning results of the three antennas/receivers onboard the vessel BALTIC TAUCHER II during the measurement campaign in the Baltic Sea, 2 October 2015 at UTC 6:28–8:18: (**a**) distance of the BALTIC TAUCHER II to the jammer on board the vessel AARON; (**b**) maximum receiver reported CN0 of the tracked satellites; (**c**) total number of tracked satellites; (**d**) geometric dilution of precision (GDOP); (**e**) horizontal positioning error of GPS SPP (when GPS solution available, i.e., N>4) (HPE); (**f**) reference trajectory of BALTIC TAUCHER II (black line) and GPS SPP results of the three antennas + receivers in the local navigation frame of vessel AARON.

Figure 9 provides an overview of GPS Single Point Positioning (SPP) results for the analyzed time frame of nearly two hours for all three antennas/receivers on board the BALTIC TAUCHER II. During this time, the vessel BALTIC TAUCHER II passed the vessel AARON (the vessel with the jammer installed) several times with increasing passing distances, as shown Figure 9a. For the entire time, except during the turning maneuvers, the PPD jammer was activated. The idea behind the deactivation of the jammer during the turning maneuvers was to start each passing of the jammer under undisturbed and, therefore, repeatable conditions. Although the turning maneuvers were performed at a distance of more than three kilometers, the on/off switching of the jammer is clearly visible from the reported maximum CN0 values, as shown in Figure 9b. When the jammer was switched off, between 9 and 11 GPS satellites have been tracked, as is easily seen in Figure 9c. At the points of maximum separation between the two vessels, the horizontal positioning error of approximately 2 m had been achieved with geometric dilution of precision (GDOP) values between 1 and 2 for all three antennas and receivers. Therefore, with the sufficient separation from the jammer, the positioning performance lies in the expected range of non-augmented code-based SPP. Note that the zone where pure GPS-positioning is prevented by the jammer is not easy to clearly define (see Figure 9f). The places where GPS positioning is possible already vary significantly between the three antennas. Additionally, a dependency on whether one is approaching the jammer or is veering away is observed. Obviously, in the former case, the GPS position solution is available on a smaller distance to the jammer when compared to the latter one. This, however, is not too surprising due to the signal acquisition and tracking mechanisms, as implemented by a typical commercial receiver. Usually, keeping track of a satellite can be performed under CN0 conditions inferior to those of the acquisition of a new satellite. Moreover, a relatively large variability in the impact of the jammer on the CN0 value can also be found in Figure 9b. The observed behavior can be, at least to some extent, explained by the specific experimental conditions. The jammer was mounted on the vessel AARON at a height comparable to the GPS antenna on the test vessel. As the jamming signal was coupling into the antenna at nearly zero degree elevation, the impact of the jamming signal strongly varied with the elevation angle (≈0.25dB/${}^{\circ }$). During the experiment, the Baltic Sea was quite rough (wave heights between 1 and 2 m) so that the vessel was rolling and pitching significantly. The variations in roll and pitch angles of the vessel translate directly to the variations in the effective elevation of the jammer with respect to the GPS antenna body frame and, hence, into the variations of the power of the injected jamming signal. Besides this, the mounting of the GPS antenna, which was not on the topmost position of the vessel (see Figure 4), could cause shadowing effects of the jamming signal by structures of the vessel itself depending on the trajectory segment.

**Figure 10:**
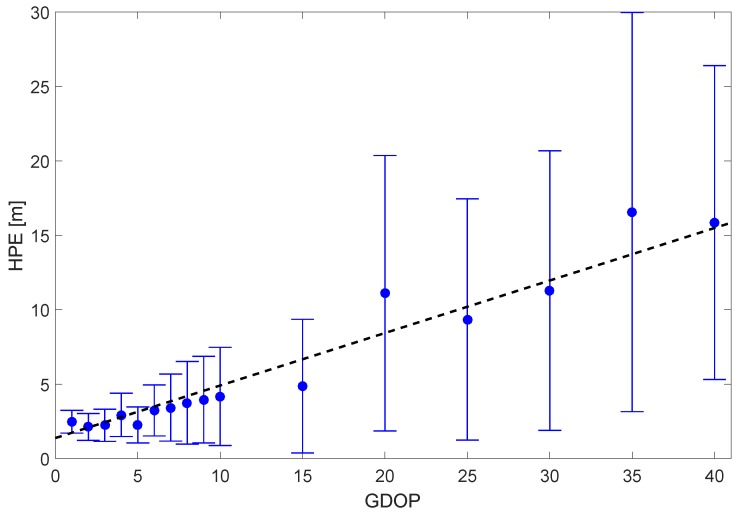
Mean HPE versus GDOP for all three antennas together. Error bars mark the 1σ values.

A careful analysis of Figure 9d,e reveal that large position errors seem to happen only when GDOP is large (i.e., GDOP >5). This could also be confirmed by analyzing the dependency of the HPE on the GDOP during these two hours. In Figure 10, the mean HPE is plotted with the GDOP. The statistics here include all measurements from all three antennas during the analyzed 2 h time span. In order to account for the fact that large GDOP values are less frequently observed, the bin size for large GDOP values has been enlarged accordingly. In Figure 10, a relatively good correlation between HPE and GDOP can be found. This also confirms the observation of the lab experiment in the first part of this section, where we have demonstrated that the impact of the applied PPD jammer on the employed geodetic receiver is mainly caused by the reduction of the number of tracked satellites. A reduced number of satellites leads to a worse satellite geometry (larger GDOP), and this correspondingly causes larger positioning errors even in the case of the same baseline noise level on each link. Although the noise level on the range measurements is also affected by the presence of the jammer, this effect can be considered minor compared to that caused by a worsened GDOP.

#### 5.2.2. Tightly and Loosely Coupled UKF Results

After the discussion of the GPS single point positioning results in the last paragraph, now the results of both loosely and tightly coupled UKF using GPS, IMU, and Doppler velocity log (DVL), as introduced in Section 3, will be analyzed. In this section, we focus on positioning results using the constant noise model as the simplest possible measurement model for the GPS measurements, with a comparison of the results of the different noise models provided afterward.

**Figure 11:**
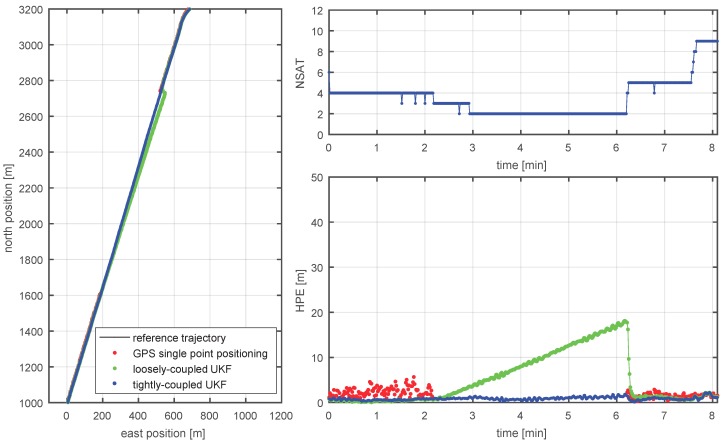
An example of the performance of the hybrid IMU/GNNS/DVL system during the time when fewer than four satellites are available: segment overview (**left**); number of available satellites (**top right**); and the HPE (**bottom right**).

In Figure 11, the positioning results are presented for an example of 8 min time span with >3 min when no SPP solution can be obtained (portside antenna, around 6:50 UTC (see Figure 9), as the number of satellites drops temporarily below four due to the proximity to the jammer. The GPS outage starts after a segment with an already relatively bad GDOP due to a decreased number of satellites, as can be seen from the HPE for the pure GPS SPP solution between t=1 and t=2 min. Both tightly and loosely coupled UKFs show good smoothing behavior within the first 2 min of that segment and keep their horizontal positioning error significantly below 2 m. Within the time interval, when the number of satellites drops below four, the loosely coupled UKF starts to drift away linearly, while the tightly coupled UKF still nicely follows the vessel track, keeping the HPE below 2 m. This example is an excellent demonstration of a great advantage of a tightly coupled architecture when compared to a simpler loosely coupled technique in a real application scenario, where the pseudorange measurements are exploited even in the case where fewer then four satellites are available. For the loosely coupled UKF, the drift during the GNSS outage is apparently linear in time. This is expected when a classical GNSS/IMU integration is augmented with the velocity measurements (DVL can be seen as an odometer). This proves to be a great advantage of DVL augmentation when compared to a more classical IMU/GNSS approach, where inertial sensors typically show position drift to be cubic in time.

**Figure 12:**
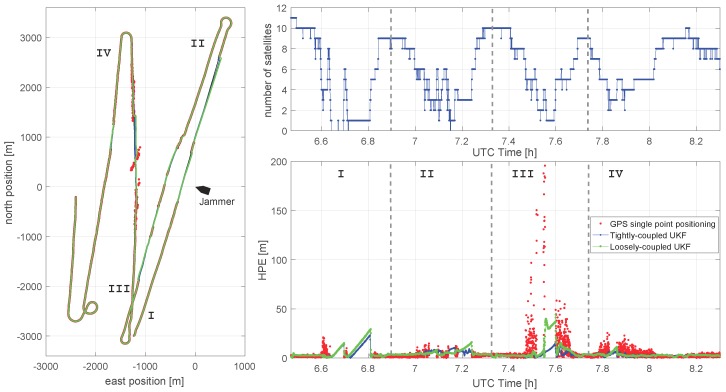
The performance of the hybrid IMU/GNSS/DVL system during the complete measurement scenario using the midship GPS antenna: overview (**left**); number of available satellites (**top right**); and the HPE (**bottom right**).

In Figure 12 the positioning results are shown for the complete scenario (approximately 2 h). In order to evaluate the UKF with the most challenging scenario, we have used here the GPS measurements from the midship antenna, which showed the largest GPS single point positioning errors (see Figure 9). During the first passing of the jammer, both tightly and loosely coupled filter showed a comparable performance with the number of the tracked satellites dropping very fast to one or even to zero satellites. In this challenging case the position solution of a tightly coupled UKF also shows a linear drift exactly due to the same reason as the loosely coupled UKF described above. Apparently, an available single range measurement is not sufficient to constrain the estimated position of the filter. This could be, at least to some extent, explained by the fact that both the position and the receiver clock offset have to be estimated. An interesting idea for a future measurement campaign would be to investigate the performance gain of a tightly coupled UKF when a more stable Rubidium oscillator is used as the reference of the receiver’s clock.

During the second jammer passing, the number of tracked satellites varies several times around four, with a maximum of 6 and a minimum of 0 satellites. Here, the tightly coupled filter still shows some advantages, although the loosely coupled filter performs reasonably well.

The largest difference between the filters can be observed during the third passing of the jammer. During that time span with a GPS outage, for a short period of time, four satellites were tracked. The GDOP for this four satellite constellation is larger than 50 (outside the scale of Figure 9d), and the largest positioning errors (HPE ≈ 200 m) occur. Here, the positioning error of the tightly coupled filter clearly stays below 20 m, while the loosely coupled filter results in positioning errors up to 50 m. The advantage of the tightly coupled filter is clearly visible. In the loosely coupled filter regime, the uncertainty in state estimate is growing during the time without GPS position updates; therefore, the new GPS positions, containing the large errors, will lead to a relatively high weight in the Kalman gain. In contrast to that, for the tightly coupled filter for almost all times, pseudorange measurements from at least two satellites are available and limit the uncertainty in the filter state. Therefore, the tightly coupled filter performs better in this challenging scenario. For the fourth passing of the jammer, the performance of both filters is roughly comparable with slight advantages of the tightly coupled configuration.

The linear drift of the tightly coupled UKF and loosely coupled UKF for the starboard and midship antenna (see Figure 11 and Figure 12), when no GPS measurements are available, seem to be mainly induced by a rather constant course over ground (COG) error to the starboard side. Estimating the COG error, which causes an 18 m drift over a time period of approximately 4 min, assuming a velocity of approximately 5 m/s, results in $\Delta COG\approx 0.8^{\circ }$. Possible root causes for this COG error are random noise measurement errors in the GNSS compass baseline vector determination, misalignment of the GNSS compass, measurement errors of the Doppler velocity log (DVL), and modeling errors and possible misalignment of the DVL. Because of the observed rather constant COG error, the contributions from the misalignment, of both the GNSS compass and the DVL with respect to the ship body frame, require further investigation. For a GNSS compass with a relatively short baseline of 1.2 m, as used in the described setup, an error of the surveyed position of the GNSS antennas of 2 cm could potentially lead to the observed $0.8^{\circ }$ error. For the DVL, the alignment error directly translates into a COG error. Note that, to display the actual vessel speed (the main application of the DVL), a misalignment of $1^{\circ }$ is fully acceptable. Nevertheless, it could have a significant impact on the positioning solution in a hybrid navigation system. Although the constant COG error can be removed by a manual misalignment adjustment, further options could include a simultaneous estimation of the misalignment parameters along with the kinematic state of the ship. Although conceptually beautiful, this approach is not guaranteed to converge, especially bearing in mind fairly limited dynamics of the vessel in typical application scenarios. Nevertheless, the achieved position performance of the tightly coupled UKF in the presented measurement campaign, where the PPD jammer was passed several times in varying distances, with HPE ≤ 30 m can be considered sufficient for basic maritime applications.

#### 5.2.3. Comparison of Different Weighting Models

The UKF results as presented above were all processed using a constant noise variance model for the GPS measurements. Here we will compare the positioning results of the tightly coupled solutions using different pseudorange variance models. For this purpose, we have processed the same measurement data three times using the GPS measurements from the midship antenna. The results of the constant noise error model are the ones from Figure 12. In Table 1, the positioning performance of the tightly coupled UKF using the three different noise models is compared. Only small differences between the models are found, as all the three models perform reasonable well. The elevation depended noise model shows the best results, especially in the maximum HPE. The results of the constant noise model and the CN0 weighting model are almost identical. These results support the results from the lab experiment of Section 5.1. The rather small differences in the positioning results of the three models are surprising. This might result from the fact that, due to the usage of a FOG IMU combined with the DVL, the filter is remarkably robust and therefore is less sensitive to the GPS pseudorange measurements.

## 6. Summary

In this paper, a hybrid navigation system is introduced using GNSS, inertial, and DVL measurements. For the first time, such a hybrid navigation system was evaluated in a real maritime jamming scenario. For the measurement campaign conducted in the civilian jamming testbed in the Baltic Sea, a Personal Privacy Device (PPD) jammer was installed on a moored vessel. A second vessel, equipped with three separately placed GNSS antennas and receivers, an inertial measurement unit (IMU), and a Doppler velocity log (DVL) passed the jammer several times with varying distances. The influence of the jammer on the three antennas varied significantly, with the GPS single point positioning being partially disrupted up to a distance of approximately 3 km and corresponding positioning errors up to 200 m.

A successful usage of GPS pseudorange measurements under challenging jamming conditions is only possible if the measurements still follow assumed test statistics within the sensor fusion algorithm. In order to determine the pseudorange error statistic under a jammed condition, a lab experiment was performed, where the output of a GNSS antenna was merged with the signal of an attenuated PPD jammer before being fed to a GNSS receiver. The analysis of this experiment shows that, as expected, the carrier-to-noise density (CN0) decreases with increasing jamming power, and the number of tracked satellites is therefore significantly reduced. Unexpected is the fact that the standard deviation of the pseudorange error is only slightly increased by the jammer. A CN0-based variance model, which is typically assumed to be the best model, substantially overestimates the error in the presence of a jammer. Out of this analysis, a standard elevation-based noise model or even a constant noise model appear to be good candidates for the application in the sensor fusion scheme.

The evaluation of the proposed IMU/GNSS/DVL UKF within the challenging jamming scenario confirmed the superior performance of the tightly coupled approach when compared to the loosely coupled scheme. Here, the advantage of the tightly coupled UKF is mainly due to a direct usage of the measurements in the segments affected by the jamming, where the number of tracked satellite drops partially below four. Due to the usage of the DVL, the filter shows only a linear position drift during complete GPS outages. This can be seen as a substantial advantage when compared to classical GNSS/IMU integration, where the positioning error grows cubically in time due to triple integration of the gyroscope errors in a strapdown navigation system. The analysis of the different weighting schemes for the GPS pseudorange measurements inside the UKF yielded rather small differences between the approaches. The best performance was achieved using the elevation-based variance model and, therefore, confirms the results of the lab experiment. The maximum horizontal position error of less than 30 meters of the tightly coupled IMU/GNSS/DVL UKF for the challenging jamming environment is small enough to successfully support most of the maritime applications.

Future work will deal with the reduction of the observed constant linear drift of 5 m/min by trying to align better the DVL and GNSS compass or, alternatively, considering a constant alignment error within the estimator. Additionally, the simultaneous usage of the measurements from all three antennas and the performance of the filter with available stable reference clock will be explored. With respect to the pseudorange error model in the vicinity of jammer, we will extend the lab experiment in order to calibrate an error model based on both the elevation and carrier-to-noise density ratio. Finally, an appropriate error model for the GNSS Doppler measurements needs to be developed.

## Figures and Tables

**Table 1:** Positioning results of the tightly coupled UKF using different noise models.

Noise Model	95% HPE [m]	99% HPE [m]	Max. HPE [m]
Elev. model	10.1	17.7	23.3
CN0 model	10.6	21.2	27.0
Const. noise	10.6	21.2	26.9
